# Alterations in the mechanical, chemical and biocompatibility properties of low-cost polyethylene and polyester meshes after steam sterilization

**DOI:** 10.1007/s10029-020-02272-w

**Published:** 2020-09-25

**Authors:** Reiko Wiessner, R. Lorenz, A. Gehring, T. Kleber, C. Benz, M. Sander, D.-U. Richter, M. Philipp

**Affiliations:** 1 Department of General and Visceral Surgery, Bodden-Kliniken Ribnitz-Damgarten, Sandhufe 2, 18311 Ribnitz-Damgarten, Germany; 2 3+ Chirurgen, Berlin-Spandau, Germany; 3 Department of Vascular Surgery, Helios Klinikum Bad Saarow, Bad Saarow, Germany; 4 Institute of Structural Mechanics (StM), University of Rostock, Albert-Einstein-Str. 2, 18059 Rostock, Germany; 5 Department of Obstetrics and Gynecology, University of Rostock, Rostock, Germany; 6 Department of General, Visceral, Vascular and Transplantation Surgery, University of Rostock, Rostock, Germany

**Keywords:** Inguinal hernia repair, Low cost mesh, Sterilization, Shrinking, Biocompatibility

## Abstract

**Introduction:**

In Africa and other Low Resource Settings (LRS), the guideline-based and thus in most cases mesh-based treatment of inguinal hernias is only feasible to a very limited extent. This has led to an increased use of low cost meshes (LCMs, mostly mosquito meshes) for patients in LRS. Most of the LCMs used are made of polyethylene or polyester, which must be sterilized before use. The aim of our investigations was to determine changes in the biocompatibility of fibroblasts as well as mechanical and chemical properties of LCMs after steam sterilization.

**Material and methods:**

Two large-pored LCMs made of polyester and polyethylene in a size of 11 x 6 cm were cut and steam sterilized at 100, 121 and 134 °C. These probes and non-sterile meshes were then subjected to mechanical tensile tests in vertical and horizontal tension, chemical analyses and biocompatibility tests with human fibroblasts. All meshes were examined by stereomicroscopy, scanning electron microscopy (SEM), LDH (cytotoxicity) measurement, viability testing, pH, lactate and glycolysis determination.

**Results:**

Even macroscopically, polyethylene LCMs showed massive shrinkage after steam sterilization, especially at 121 and 134 °C. While polyester meshes showed no significant changes after sterilization with regard to deformation and damage as well as tensile force and stiffness, only the unsterile polyethylene mesh and the mesh sterilized at 100 °C could be tested mechanically due to the shrinkage of the other specimen. For these meshes the tensile forces were about four times higher than for polyester LCMs. Chemical analysis showed that the typical melting point of polyester LCMs was between 254 and 269 °C. Contrary to the specifications, the polyethylene LCM did not consist of low-density polyethylene, but rather high-density polyethylene and therefore had a melting point of 137 °C, so that the marked shrinkage described above occurred. Stereomicroscopy confirmed the shrinkage of polyethylene LCMs already after sterilization at 100 °C in contrast to polyester LCMs. Surprisingly, cytotoxicity (LDH measurement) was lowest for both non-sterile LCMs, while polyethylene LCMs sterilized at 100 and 121 °C in particular showed a significant increase in cytotoxicity 48 hours after incubation with fibroblasts. Glucose metabolism showed no significant changes between sterile and non-sterile polyethylene and polyester LCMs.

**Conclusion:**

The process of steam sterilization significantly alters mechanical and structural properties of synthetic hernia mesh implants. Our findings do not support a use of low-cost meshes because of their unpredictable properties after steam sterilization.

## Introduction

With up to 20 million operations per year, inguinal hernia repair is one of the most frequently performed operations in general surgery worldwide [1]. Almost one third of all men and about 3% of all women can develop an inguinal hernia during their lifetime [2]. The prevalence of inguinal hernia is high in low income countries (LICs). Because the health care system in LICs is mostly underdeveloped and elective hernia repair is rare. Most repairs are performed as emergencies; the resulting mortality is as high as 40% [3, 4]. In addition, there are significantly more scrotal hernias in LICs than in higher-income countries (HICs), as most patients undergo surgery late. In these cases, a pure-tissue technique is often not feasible [5, 6]. Large hernia defects lead to the necessity of synthetic mesh reinforcement. On the one hand, these are unaffordable for large parts of the population, and on the other hand the implantation techniques often have not been learned by the few surgeons available in LICs [7–10].

The current HerniaSurge Guidline also focuses on the problem of surgery of inguinal hernias in LRSs [2]. The recommendations of the HerniaSurge Guidline apply to every patient worldwide. For most of the inguinal hernias the Lichtenstein-Technique with use of Low Cost Meshes under local anesthesia was recommended. The chemical and physical properties of the LCMs should be known.

While the studies carried out on patients show equivalent results in comparison to commercial meshes (CMs) [5, 11–13], other studies show inadequate results of the different LCMs after steam sterilization [14]. The LCMs from Ethiopia, Ghana and India tested by Mitura et al. shrink massively after sterilization at 121 °C and could therefore not be recommended for use in patients [14].

The aim of this work was to investigate the influence of steam sterilization at different temperatures on the mechanical and chemical properties as well as the biocompatibility of fibroblasts in two LCMs made of polyethylene and polyester.

## Material and methods

### Material

Two mosquito meshes made of polyethylene (Amsa Plastic, India) and polyester (Brettschneider Moskitonetze, Germany) were used for the mechanical, chemical and biocompatibility tests.

- **Low cost mesh** made of **polyethylene**, large-pored (1.5 × 1.9 mm), monophilic, lightweight (53.7 g/m^2^) polyethylene mesh (Amsa Plastic, India). The mesh was kindly provided by Jessica Beard (M.D., M.P.H., Temple University, Philadelphia, USA).
- **Low cost mesh** made of 100% polyethylene terephthalate (**polyester**), large-pored (1.4 × 1.9 mm) with a weight of 30 g/m^2^ (Brettschneider Moskitonetze, Germany).

**Fig. 1:**
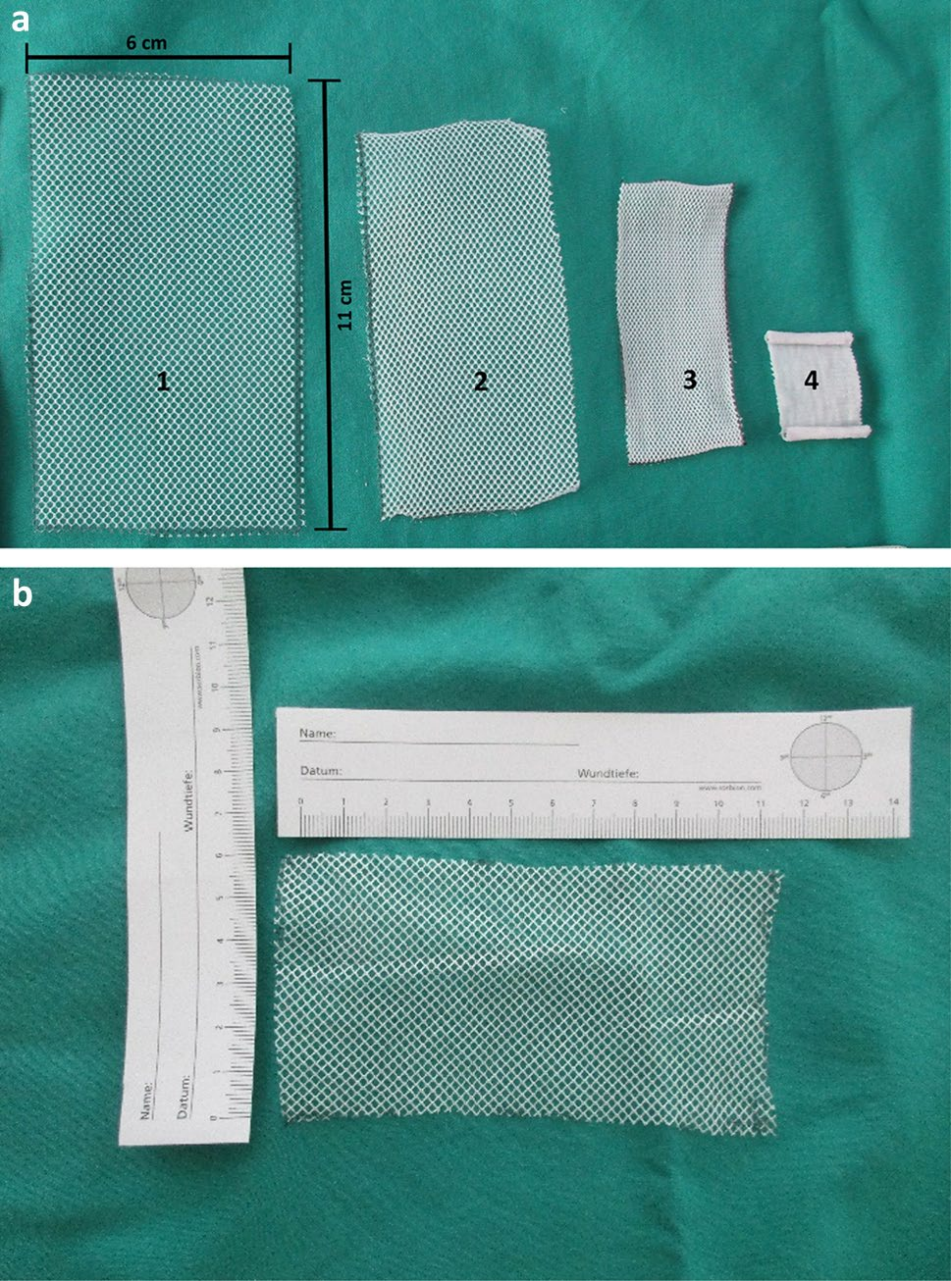
Photograph **a** shows the 6 × 11 cm polyethylene mesh unsterile (**1**), after sterilization at 100 °C (**2**), 121 °C (**3**) and 134 °C (**4**). A significant shrinkage occurs already at 100 °C horizontally and vertically. At 134° C, no grid structure can be detected. Picture **b** shows a polyester (mosquito) mesh of 6 × 11 cm after sterilisation at 134 °C

The LCMs were cut to the size of 11 × 6 cm to represent the average implantation size of a mesh when using the Lichtenstein hernioplasty technique (Fig. 1a, b). The mosquito meshes were sterilized by steam sterilization at 100 °C (29 min), 121 °C (18 min) and 134 °C (5 min). For the 100 °C sterilization we used a steam sterilizer (NübyTM, Natural TouchTM, Monroe, Louisiana, USA), which is conventionally used for sterilizing baby bottles. This steam sterilization was also intended to represent, among other things, the situation of sterilization with limited resources (boiling of instruments as in the nineteenth century, occasional power failures) in remote regions in Africa or other LICs. The use of non-sterile polyester and polyethylene meshes as a control was essential for our investigations to detect any material changes caused by sterilization. Since fibroblasts quickly contaminate in a non-sterile environment, the surrounding environment (nutrient medium) was mixed with antibiotics/antifungals. The unsterile meshes were also treated with an antibiotic and an antifungal agent. Penicillin/Streptomycin (100 × dilution; Sigma-Aldrich) was used as an antibiotic and Amphotericin B (250 µl/ml; PAN Biotech GmbH) as an antimycotic.

The following results are shown in the diagrams and figures as follows:

1. Polyethylene non-sterile
2. Polyethylene 100 °C
3. Polyethylene 121 °C
4. Polyethylene 134 °C
5. Polyester unsterile
6. Polyester 100 °C
7. Polyester 121 °C
8. Polyester 134 °C

For the biocompatibility tests, 10 × 10 mm pieces of meshes were punched out of the first cut pieces of mesh and then placed in the reservoirs of a 12-well cell culture plate (Biochrom AG, Berlin, Germany) without wrinkles [15, 16].

### Mechanical testing

Tensile tests were performed for both materials: both as unsterile and also following their different conditions of sterilization. Rectangular specimens were cut from the mosquito meshes. A schematic drawing of the specimen size and the characteristic measures of the clamping facility is given in Fig. 4a. The specimens were cut from the mesh in two orthogonal orientations (*O1* and *O2*) to investigate the influence of the orientations of the fiber (mesh) structure in relation to the loading direction. At least four specimens were tested for each condition (2 × *n* = 4). A uniaxial servo hydraulic testing machine INSTRON 8800 (Instron, USA) with a total actuator stroke of 150 mm was used for the tests. The forces were measured with a HBM load cell U2A (Hottinger Baldwin Messtechnik, Germany) load cell with a load range of ± 500 N. The tests were performed in position control mode with an actuator velocity of 1 mm/min. Each specimen was clamped with the same clamping pressure, since the screws of the clamping mechanism were tightened at the same moment. Additionally, the tests were documented with an industrial 5MP monochromatic camera (isi-sys GmbH, Germany) which was controlled by the software VIC-Snap (correlated solutions, USA).

### Chemical analyses

The chemical composition of the meshes was identified using differential scanning calorimetry, microtome section and infrared (IR)-spectroscopy. Sample preparation: the meshes were cut to a size of 2 cm^2^ and analyzed using a headspace preparation (*n* = 1).

#### Differential scanning calorimetry

The samples were characterized for their thermal properties using differential scanning calorimetry analysis (Mettler Toledo DSC823e, Switzerland). The samples were heated under a flow of dried air from 30 to 190 °C at 10 °C/min, cooled down to 30 °C at 20 °C/min and subsequently heated for a second cycle up to 300 °C at 5 °C/min.

#### Microtome sections

Suitable microtome sections were photographed at variable magnification (10 × , 20 × or 40 × ) by means of a Leica DMLS microscope and a Leica EC3 camera (Leica Biosystems, Germany).

#### IR-spectroscopy

The infrared spectra were collected by a Nicolet 380 FT-IR spectrometer (Nicolet™, USA) with a Smart Orbit ATR diamond accessory (30,000–200 cm^−1^) at room temperature.

IR parameter:

Number of scans: 32.

Scan width: 4.000–525 cm^-1^.

Resolution: 4 cm^-1^.

### Biocompatibility research methods

We have already described in detail a large proportion of the test methods we used (Scanning Electron Microscopy (SEM), cytotoxicity/LDH, pH-value determination and glycolysis test) and would like to refer to our explanations [15, 16]. One analysis per parameter was performed (*n* = 1).

#### Fibroblasts

We cite our earlier, detailed remarks [15, 16]. To study the biocompatibility properties of the meshes, tissue-specific human fibroblasts were available. Fibroblasts synthesize the components of the intracellular substance, the matrix and the fibers. They are in the organism both in the developing and growing connective tissue, as well as in the differentiated loose connective tissue. For culturing the cells, a section of approximately 30 mm^2^ of sub-epithelial tissue from female donors was used. Each individual tissue sample was divided into three to four smaller segments and prepared with enzymes with collagenase (PAA; 3–4 h; 37 °C). Subsequently, the sample was cultivated in culture medium flasks (culture surface area 25 cm^2^, Sarstedt) until a monolayer formed. An ethics committee approval by the University of Rostock is available.

#### Stereo microscopy and scanning electron microscopy for the end-point determination

We used scanning electron microscopy and, in addition, stereomicroscopy to visualize the structures of materials. Before incubation with fibroblasts, the different LCMs were examined by means of a stereomicroscope (Stemi DV4, Fa. Zeiss, Jena, Germany). This review was carried out by co-author Dagmar-Ulrike Richter (Research Laboratory of the University Women's Hospital, University Medicine Rostock). At the end of the long-term experiment (12 weeks), scanning electron microscopy (SEM) was used to determine the endpoint [15, 16]. We refer to our earlier detailed test descriptions [16].

### Biochemical assays of the biocompatibility

It is known that biomaterials can be cytotoxic if cell damage occurs during their use. Therefore, the determination of cytotoxicity and viability is an integral part of testing for biocompatibility of materials.

#### Viability test (mitochondrial activity of fibroblasts)

The CellTiter-Glo^®^ Luminescent Cell Viability Assay (Promega Corporation, Madison USA) is a cell-based assay for the detection of cell viability. The principle is based on the measurement of the ATP content in an ATP-dependent luciferase reaction. The determined ATP content is a measure for metabolic cell activity. The conversion of luciferin by means of a recombinant luciferase (Ultra-Glo™Luciferase) produces oxyluciferin and light. The strength of the light signal is measured with a luminometer (Promega Glomax Multi Detection Microplate Reader) and is proportional to the number of living cells. The measurements were performed with the respective mesh materials after 48 h incubation. This incubation time was derived from previous pilot studies from which it is known that fibroblasts react very quickly to foreign materials.

#### Cytotoxicity testing (LDH; Roche)

In this study, the cytotoxicity was analyzed using the Roche ELISA KIT. The ubiquitous LDH is very well suited for this testing procedure, not only because of its stability in the culture medium; an additional aspect is its resistance to proteases and its sufficient quantity in the target cells. We refer to our earlier detailed test descriptions [15].

### Metabolism of the cells

#### pH value determination

The pH value analyses were performed with the ORION 3 STAR electrode (Fa. Thermo Scientific) in the cell culture supernatant. The pH reference value corresponded to the pH value of the pure culture medium (medium + fetal calf serum + antibiotics). After the addition of cells and wetting samples, a pH value difference of 0.30–0.45 arose from medium change to medium change, which is caused by metabolic processes in the cells. This pH value change remained constant the entire time. The measurement was performed after 48 h incubation time. Here we refer to our earlier, detailed remarks [16].

#### Glycolysis

The determination of the extracellular glucose content in the cell culture supernatant is a measure for the glycolytic degradation of glucose in the cells. If the glucose content is lowered, this indicates that the cell's metabolism is good. If the cell is decomposed, there is an increase in glucose in the cell culture supernatant. The glucose analyses during the network contact provide an indirect indication of cell vitality. Here we refer to our earlier, detailed remarks [16].

#### Lactates

Lactate is the salt of lactic acid and is formed when, during intensive exercise, contact is made with mesh material. In this case, the oxygen absorbed via cell respiration is no longer sufficient to cover the energy requirement. This means that the normal aerobic metabolism is no longer sufficient to produce energy, and consequently, anaerobic metabolism increases (glycolysis). This then results in increased lactate.

##### Principle: enzymatic LOD method

L-lactate is oxidized by lactate oxidase (LOD) to pyruvate and hydrogen peroxide (H2O2). The reaction of peroxidase (POD), hydrogen peroxide (H2O2), 4-aminoantipyrine (4-AA) and a hydrogen donor (H-donor) produces a colored product. The intensity of the color is proportional to the lactate concentration.

1. $${{\rm L}} - {{\rm lactate }} + {{\rm O}}_{2} {\longrightarrow}{{{{\rm LOD}}}}_{{\rm pyruvate}} + {{\rm H}}_{2} {{\rm O}}_{2}$$
2. $${\text{H}}_{2} {\text{O}}_{2 } + 4 - {\text{AA}} + {\text{H}} - {\text{donor}} {\longrightarrow}_{{{\text{POD}}}} {\text{chromogen}} + 2{\text{ H}}_{2} {\text{O}} $$

Just like the glucose determination, lactate analyses were carried out in cell culture supernatants. Beckman Coulter (Beckman Coulter GmbH; Krefeld; Germany) was used for the lactate measurements. Double determinations were also performed here. As with glucose, the lactate analysis was performed after 48 h incubation time.

## Results

### Macroscopy and microscopy

**Fig. 2:**
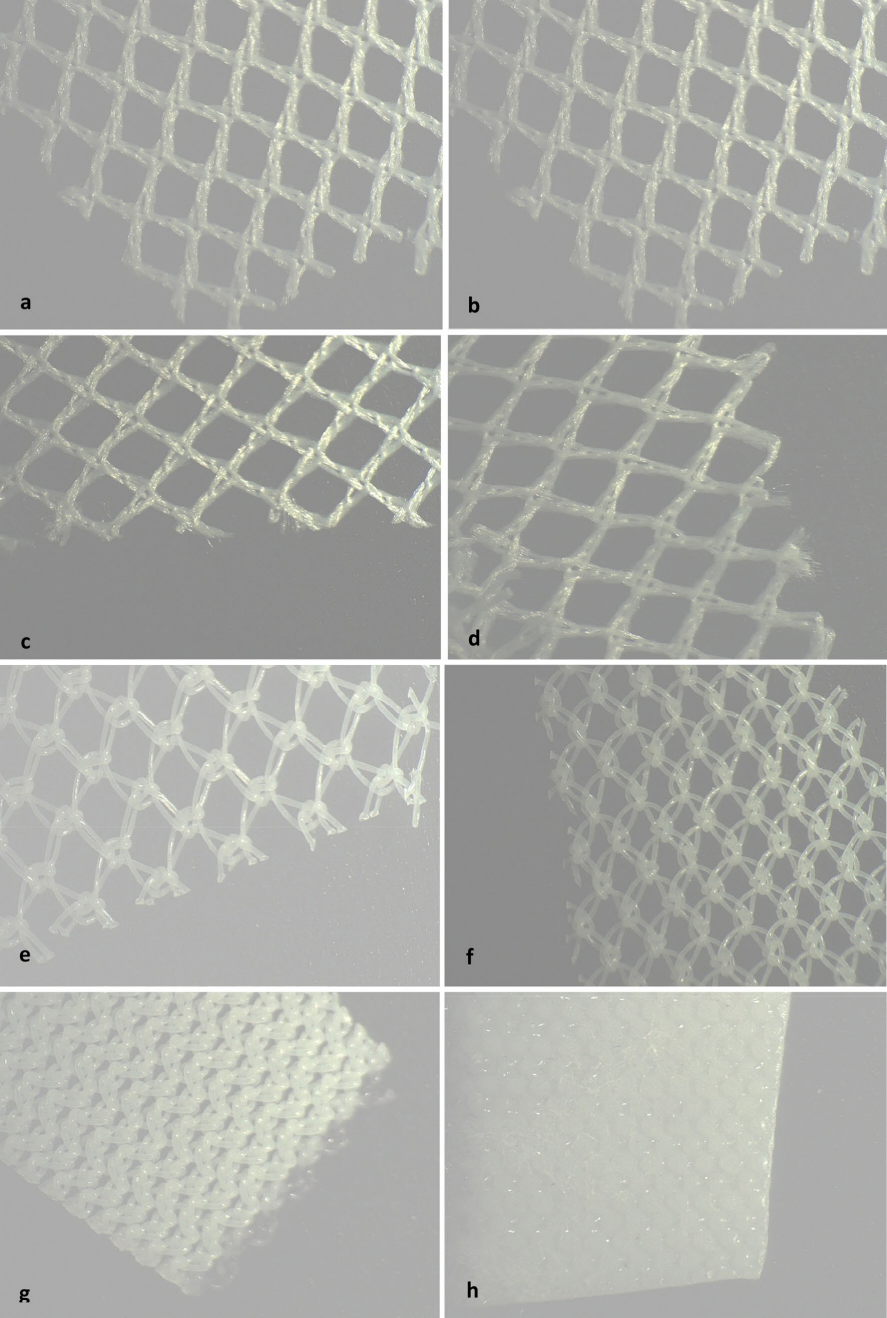
Stero microscopy (40 × magnification) of polyester LCMs: **a** unsterile; **b** sterilized at 100 °C; **c** at 121 °C; **d** at 134 °C. Stero microscopy (40 × magnification) of polyethylene LCMs: **e** unsterile; **f** sterilized at 100 °C; **g** at 121 °C; **h** at 134 °C. *LCMs* low-cost-meshes

Even macroscopically, a shrinkage of the polyethylene LCMs after steam sterilization can be observed (Fig. 1a). The polyethylene mesh showed a slight shrinkage of the meshes by about 1/3 in width and 1/5 in length even at only 100 °C. At 121 °C the polyethylene mesh structure is still visible, but extremely deformed. The meshes have shrunk considerably. It has a stiff and polygonal consistency. At 134 °C the mesh structure is no longer visible (Fig. 1a, 2 h). On the other hand, the macroscopy and stereomicroscopy at 40 × magnification showed no structural changes in the polyester LCMs after sterilization compared to the unsterile mesh (Fig. 1b and 2a ,b, c, d).

**Fig. 3:**
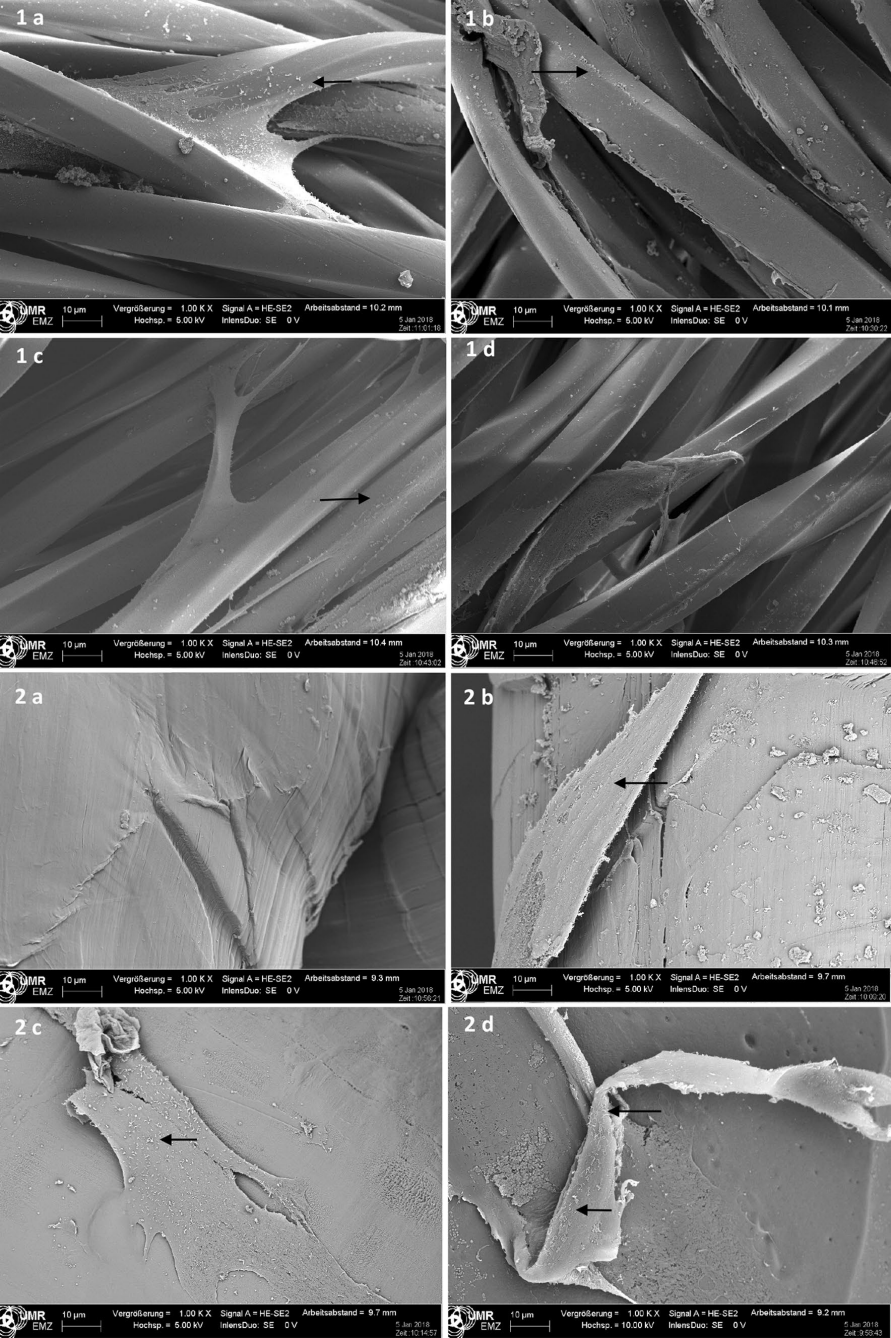
Scanning electron microscopy 12 weeks after incubation with fibroblasts at polyester (**1**) and polyethylene (**2**) LCMs. **1**. **a** unsterile; **b** sterile at 100 °C; **c** sterile at 121 °C; **d** sterile at 134 °C. **2. a** unsterile; **b** sterile at 100 °C, **c** sterile at 121 °C. a **d** sterile at 134 °C . *LCMs* low-cost-meshes

The stereomicroscopy of unsterile and sterilized (100 °C) polyethylene LCMs showed a good mesh structure with a slight shrinkage (Fig. 2e, f). After sterilization at 121 °C the mesh structure is still visible, but extremely deformed and has shrunk considerably. It showed a distinct alteration of the fiber-texture (Fig. 2g). After sterilization at 134 °C the mesh structure is no longer visible and the fibers agglutinated. The haptic aspect of the mesh is a rigid consistency with sharp edges (Fig. 2h).

The SEM of the polyester LCM showed a moderate, heterogeneous growth of fibroblasts on all meshes, independent of the sterilization procedure. A complete closure of the meshes by proliferating fibroblasts could not be detected 12 weeks after incubation. All polyester LCMs showed a good thread structure with moderate heterogeneous growth of fibroblasts. The unsterile and the polyethylene LCM sterilized at 100 °C showed a delicate, thin fibroblast growth. The polyethylene LCMs sterilized at 121 and 134 °C showed a very thin growth of fibroblasts while the net structure was lifted (Fig. 3).

### Mechanical testing

#### Polyester

##### Deformation and damage

**Fig. 4:**
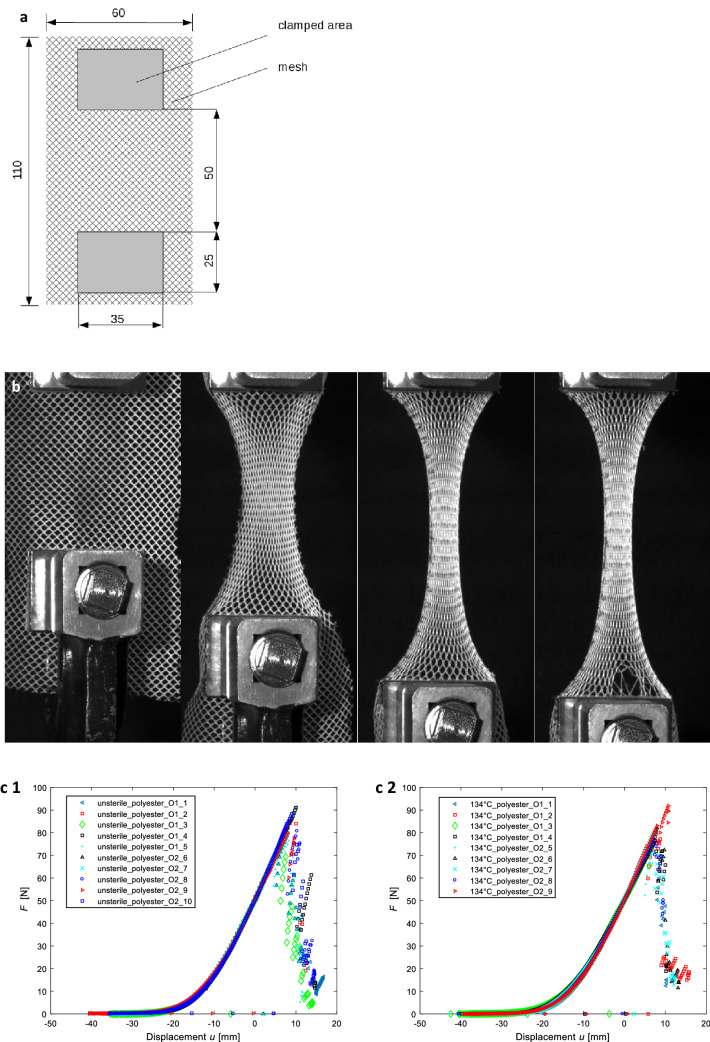

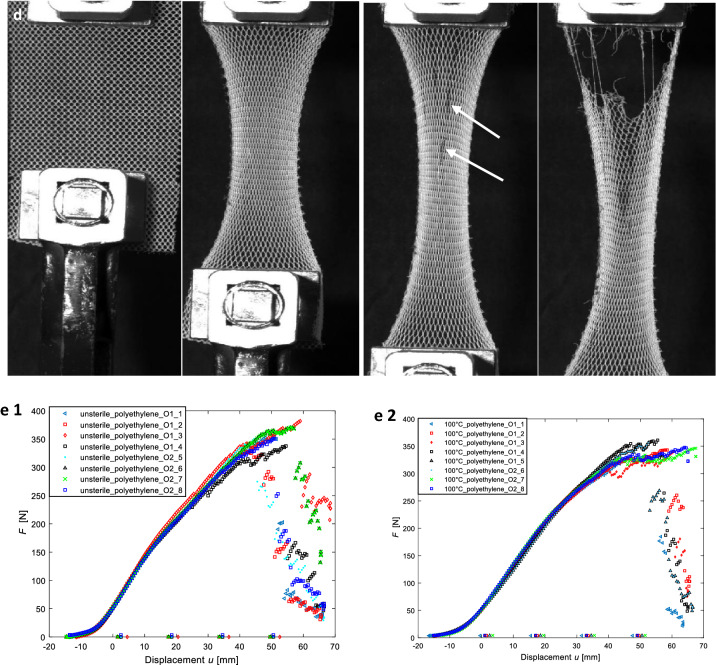
**a** Schematic illustration of the specimen and clamping at the beginning of the tensile tests. **b** Typical deformation and damage behavior in the unsterile polyester mesh. **c** Force–Displacement curves of polyester: one unsterile; two sterilized at 134 °C. (*O1* and *O2* designate two orthogonal directions in which the specimen were cut from the basic mesh) **d** Typical deformation and damage behavior in the unsterile polyethylene mesh. **e** Force–Displacement curves of polyethylene: one unsterile; two sterilized at 100 °C. (*O1* and *O2* designate two orthogonal directions in which the specimen were cut from the basic mesh)

The polyester meshes showed a significant contraction during the tests; see Fig. 4b. Since the clamping prevents the contraction, the fibers in this region are highly stressed. The failure always initiated close to the clamping region. Failure of fibers in the other regions of the specimens could not be observed macroscopically.

##### Tensile force and stiffness

Figure 4c shows the force–displacement curves for unsterilized meshes and after sterilization at 134 °C. At the beginning of the test, the force does not increase. After a few millimeters of elongation, the force increases progressively until it reaches a certain slope. The slopes of the different specimen are quite similar. It must be noted that the curves have a distinct linear region. The stiffness is quite constant until the specimen fails. The average slope/stiffness is approximately 3.7 N/mm. After reaching a maximum, the tensile forces decrease significantly due to failure of several fibers. The direction in which the specimen were taken from the basic mesh (orthogonal directions designated as *O1* and *O2* in Fig. 4c, e) showed no significant influence on the mechanical behavior. The meshes are thus equally strong, both vertically and horizontally.

Table 1 gives an overview on the variation of the maximum tensile force for all tested specimen. The sterilization at 100 °C leads to a decrease of the maximum tensile force. For the other three conditions the maximum, minimum and the arithmetic mean value do not differ significantly. However, for “100 °C” all values are approximately 10 N lower than for the other conditions. The standard deviation is significantly higher for the sterilization at 100 °C than for the others.

**Table 1:** Overview of the maximum tensile forces for all tested polyester specimens

	Unsterile	100 °C	121 °C	134 °C
Maximum value	91.1	84.8	90.8	91.9
Minimum value	74.1	60.8	73.8	72.5
Artihmetic mean	84.0	72.2	81.0	79.4
Standard deviation	5.8	8.3	6.0	5.4

All values given in [N]

#### Polyethylene

##### Deformation and damage

Two differences could be observed during the polyethylene tests in comparison to the polyester tests. First, the polyethylene specimen does not contract as much as the polyester specimen during the tests. Second, the initiation of failure is not only limited to the clamping region (compare Fig. 4d, arrows) from a macroscopic point of view. However, the final failure of the specimen is also based on the failure of multiple fibers in the clamping region.

##### Tensile force and stiffness

Figure 4e shows the force–displacement curves for the tests of the unsterile specimen and the sterilized specimen at 100 °C, respectively. The curves of both diagrams correlate quite well. The maximum tensile forces are approximately four times higher than for the polyester specimen. Another difference in the results for polyethylene is that the slopes of the curves are not constant, but the slopes show a declining characteristic until the maximum force is reached. After the force has reached its maximum value, multiple fiber failures occur (compare Fig. 4d). However, there is a linear region for forces up to approximately 200 N. The average stiffness in the linear region is about 8.8 N/mm, which is more than twice the stiffness of the polyester specimen.

Table 2 gives an overview of the variation of the maximum tensile force for all tested specimens, where the maximum, minimum, arithmetic mean value and standard deviation for the maximum tensile force are listed. The standard deviation for the unsterile specimen is significantly higher than for the specimen sterilized at 100 °C. The minimum and the arithmetic mean values are quite similar, while the maximum value is higher for the unsterile condition.

**Table 2:** Overview of the maximum tensile forces for all tested polyethylene specimens

	Unsterile	100 °C
Maximum value	381.8	361.1
Minimum value	329.2	336.9
Artihmetic mean	356.7	347.3
Standard deviation	18.0	7.1

All values given in [N]

### Chemical testing

All meshes are produced as monolayers. The polyester LCM has a characteristic melting point for polyethylene terephthalate (PET) in a range of 254–269 °C. Therefore, no deformation (shrinkage) during sterilization is observed. The LCM made of polyethylene does not consist of low-density polyethylene but rather of high-density polyethylene with a characteristic melting point of 137 °C (Table 3). The polyethylene mesh sterilized at 134 °C was not suitable for microtome section due to massive shrinkage.

**Table 3:** Results of the chemical tests

Sample name	Deformation	Melting point/transition [°C]	Identified materials
Polyethylene unsterile	No	137	HDPF
Polyethylene 100 °C	No	137	HDPF
Polyethylene 121 °C	Yes	137	HDPF
Polyethylene 134 °C	Yes	–	HDPF
Polyester unsterile	No	256	PET
Polyester 100 °C	No	256	PET
Polyester 121 °C	No	254	PET
Polyester 134 °C	No	259	PET

**Fig. 5:**
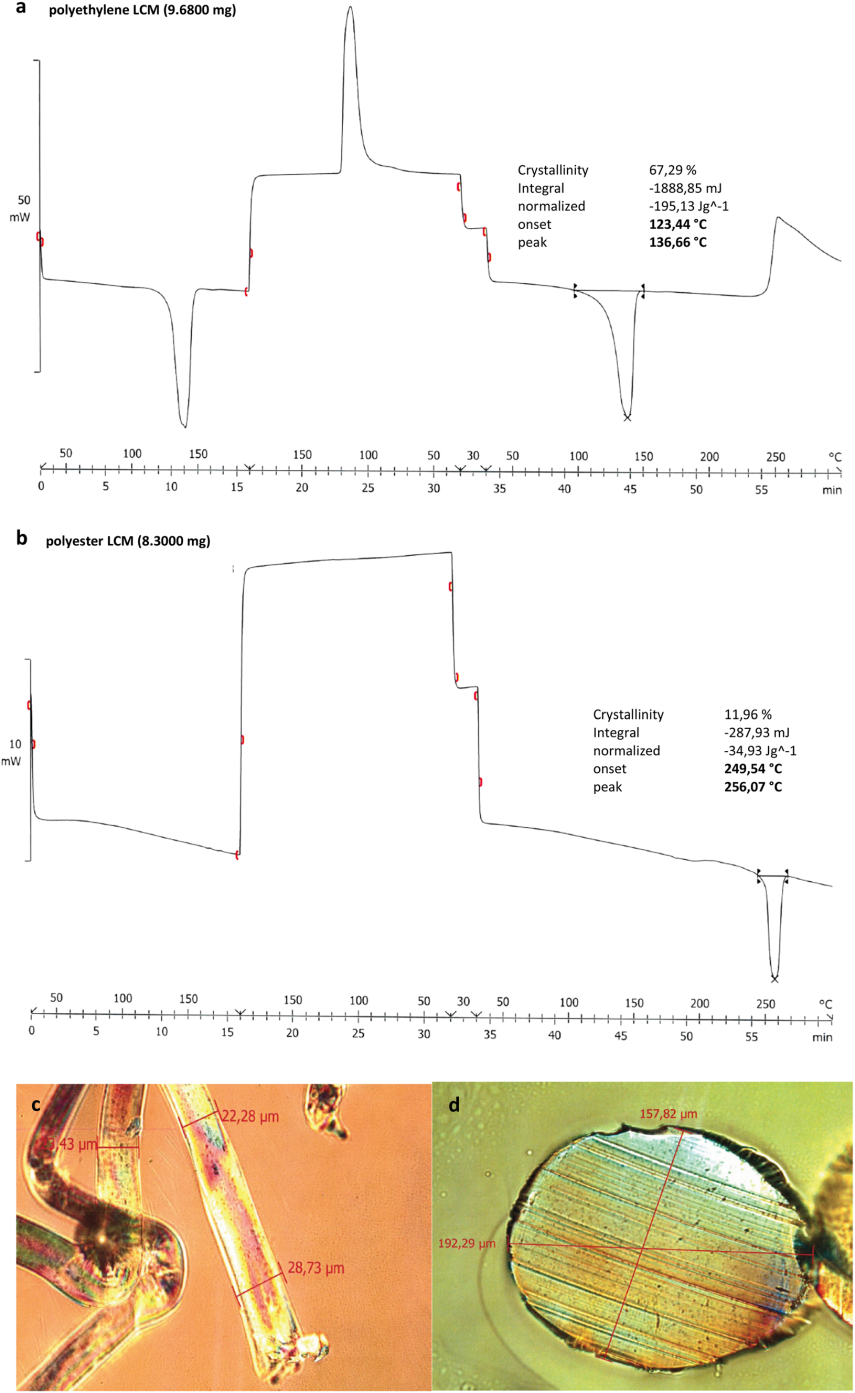
Chemical analysis. **a** differential scanning calorimetry analys of unsterile polyethylene mesh. **b** differential scanning calorimetry analysis of unsterile polyester mesh. **c** microtome section of unsterile polyethylene. **d** microtome section of unsterile polyester. **IR-analysis** of unsterile polyester (cm^−1^): *ν*(C–H) 2957 (strong), *ν*(C–H) 2955 (strong), *ν*(CH_2_) 1447 (deformation in plan). **IR-analysis** of unsterile polyethylene (cm^−1^): *ν*(C–H) 2998 (strong), *ν*(C–H) 2934 (strong), *ν*(C = O) 1722 (strong), *ν*(C–C–O) 1326 (strong), *ν*(O–C–C) 1093 (strong), *ν*(C = C) 722 (very strong)

The characteristic vibrational bands in the IR spectra confirm the chemical composition of the meshes (Fig. 5).

### Biocompatibility of fibroblasts

**Fig. 6:**
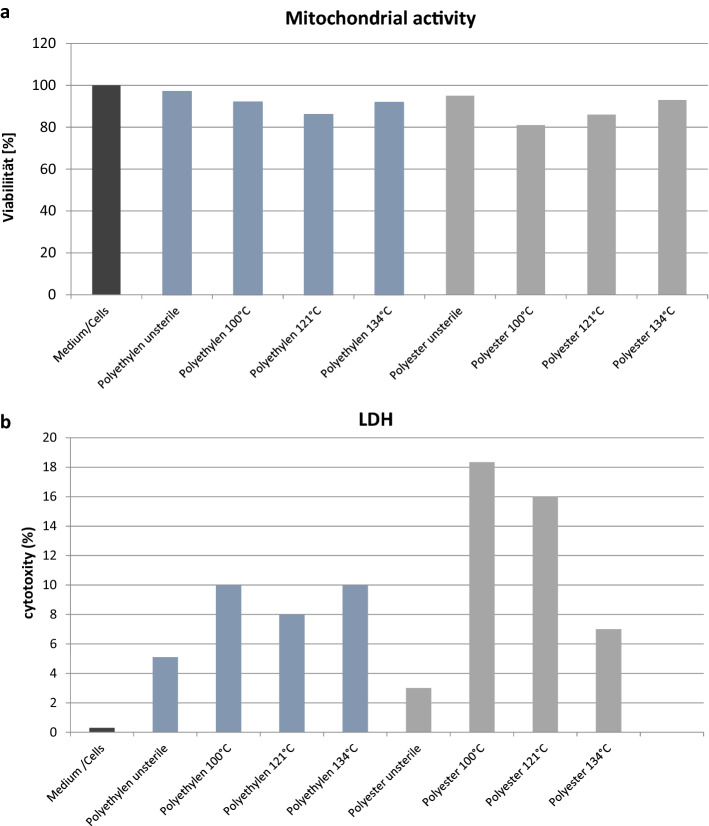
Biochemical analyses. **a** Measurement of the viability of the fibroblasts 48 h after incubation. **b** Measurement of the cytotoxicity of the fibroblasts 48 h after incubation

The measurement of the viability of the fibroblasts 48 h after incubation showed no significant changes between the unsterile and sterilised meshes. The vitality of the fibroblasts was greater than 80% in all meshes. Interestingly, compared to the unsterile meshes, both the sterilized polyester and polyethylene meshes showed a lower mitochondrial activity (Fig. 6a). The LDH measurement also showed the lowest cytotoxicity for both unsterile LCMs, while in particular the sterilized polyethylene meshes with 18.35% (polyethylene LCM 100 °C) and 16.0% (polyethylene LCM 121 °C) showed a significant increase in cytotoxicity after 48 h incubation with fibroblasts compared to the medium/cells (0.3%) (Fig. 6b).

**Fig. 7:**
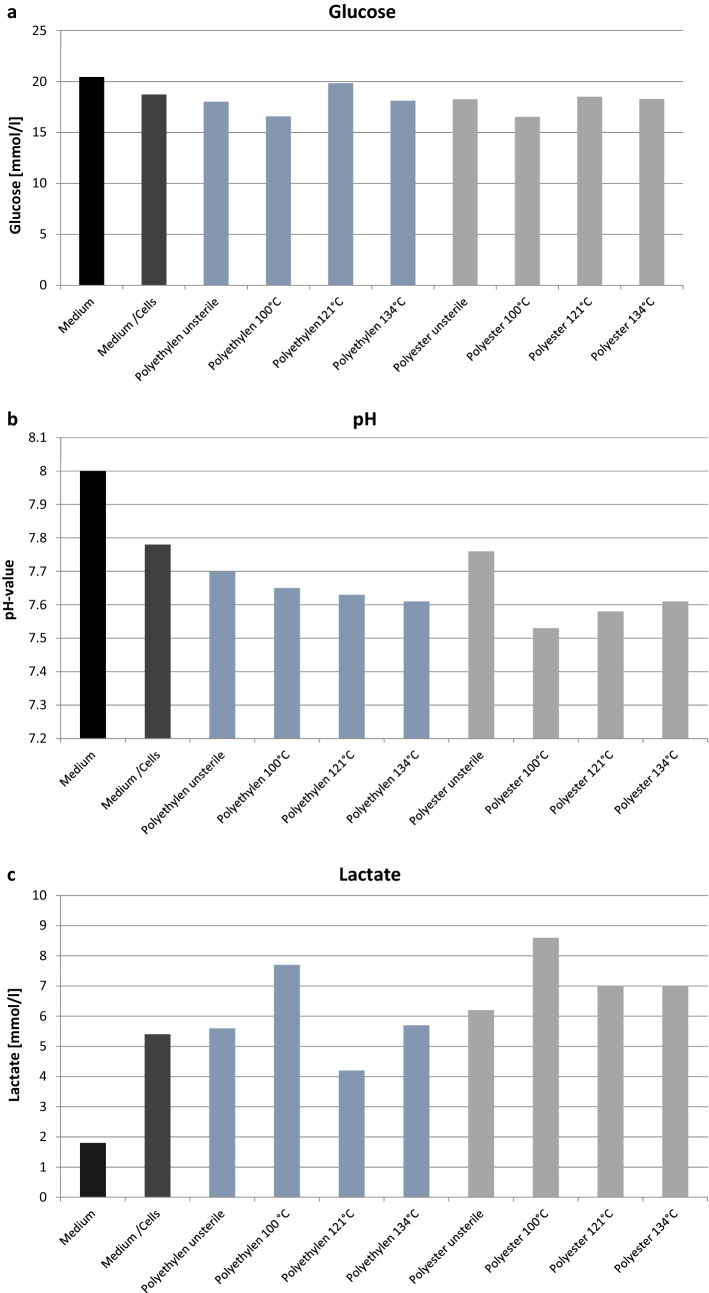
Metabolism of the cells. **a** Measurement of the glucose metabolism of the fibroblasts after 48 h of incubation. **b** Measurement of the pH value of the fibroblasts after 48 h of incubation. **c** Measurement of the lactate metabolism of the fibroblasts after 48 h of incubation

Measurement of the glucose metabolism showed normal metabolism of the fibroblasts without significant change on all unsterile and sterilized polyethylene and polyester low cost meshes (Fig. 7a). The pH value of non-sterilized LCMs (LCM polyethylene pH value 7.7; LCM polyester pH value 7.76) was the lowest in comparison to the medium/cells (pH value 7.78), where there are ideal conditions for the fibroblasts (Fig. 7b). Measurement of the lactate metabolism showed the highest lactate production in fibroblasts on polyethylene (7.7 mmol/l) and polyester (8.6 mmol/l) meshes sterilized at 100 °C (Fig. 7c).

## Discussion

The HerniaSurge Guidline recommends the use of meshes for hernias also for LICs [2]. In LICs, however, the conventional commercial meshes are unaffordable for the majority of the patients, so that due to the lack of alternatives, cost-effective alternatives were sought [11]. Tongaonkar, in particular, is considered a pioneer in the use of mosquito meshes and has shown excellent results in more than 700 patients over 10 years with a follow-up of 12–18 months [17]. Several research groups were also able to demonstrate equivalent results in comparison to CMs [5, 7, 12, 13, 18], so that the current guideline makes the (weak) recommendation for the use of LCMs in the Lichtenstein technique [2]. In the guideline, the problem of sterilization of LCMs is only briefly described [2]. However, the literature used to prepare the recommendation shows slight changes (shrinkage) in polyethylene LCMs after steam sterilization at 121 °C [19].

The only prospective randomized study does not describe any changes in low density polyethylene LCMs sterilized at 121 °C for 20 min [12]. The randomized prospective study published by Löfgren et al. with a follow-up of one year showed no differences in the clinical results (recurrence rate, p. o. complications) compared to the commercial polypropylene mesh used in the comparison group [12]. The polyethylene LCMs were cut into 10 cm × 15 cm pieces and reference was made to the studies by Stephenson and Kingsnorth, who in their publication demonstrated the minimal structural changes described above [19].

To further substantiate these results, which were obtained directly from the patient, in in-vitro experiments and, if necessary, animal experiments, we have also tried to obtain a low density polyethylene mesh, as these meshes were used most frequently in previous studies [11, 12, 17, 19]. Our aim was to prove in vitro that LCMs made of polyethylene and polyester are safe to use, as described in the introduction. Thus, our former investigations of cell proliferation, cytotoxicity, oxidative stress, pH and glycolysis including SEM did not show significant differences between the polyester LCM, which is also used currently, and various commercial meshes (inter alia Ultrapro™ (Ethicon, Norderstedt, Germany), Parietex^R^ (Medtronic GmbH, Meerbusch, Germany)) [15].

It is well known that different sterilization processes for synthetic materials also lead to very different changes in the individual polymers [20]. For example, Müller et al. demonstrated in 1999 that only γ radiation should be used to sterilize polyethylene, since sterilization with steam at 121 and 134 °C leads to deformation and destruction of polyethylene. The polyethylene used, which had a crystal melting point of 118 °C, showed pronounced changes in the fibrils even at 121 °C [20]. Sterilization with 3% formaldehyde for one hour at 60 °C also led to changes in the polyethylene sample [20]. Our results of the chemical analysis show that the polyethylene LCM examined was high density polyethylene (HDPE) and not low-density polyethylene (LDPE), as described above. HDPE is even more resistant to heat and chemicals than LDPE [21]. In their 2011 work, Stephenson et al. also investigated a mosquito mesh from India, which consisted of 50% polypropylene and 50% polyethylene [19]. Steam sterilization of the initial 7 × 5 inch (17.8 × 12.7 cm) mesh at 134 °C led to massive shrinkage, as in our investigations. Sterilization at 121 °C for 20 min resulted in a shrinkage of 30%, which is lower than in our tests (Fig. 1c, 2c) [19]. These (shrunken) meshes were then implanted in 51 patients (54 hernias) in a size of 10 × 12 cm [19]. The 6-month follow-up showed no complications [19]. A shrinkage of 30% naturally leads to a change in mesh size and thus in effective porosity. In their prospective randomized study Löfgren et al. also implanted these (shrunken) meshes in 150 patients, whereby the initial size of the meshes before sterilization at 121 °C was approx. 10 × 15 cm (with approx. 30% shrinkage after sterilization then approx. 7 × 11.5 cm; assuming the same chemical composition of the Amsa Plastic mesh used as that of Stephenson et al. 2011) [12]. For a hernia repair using the Lichtenstein technique, this mesh size is just barely acceptable. However, the mesh size is only one parameter that can change due to sterilization of synthetic materials.

Thus, the aim of our mechanical investigations was to identify the influence of different sterilization methods on the mechanical properties of two different meshes. Since the focus was on the comparison of the unsterile conditions to different sterilized specimen, the test setup was chosen to be intriguingly simple rather than to mimic a complex condition after implantation in a human body. The investigations served this purpose very well. It was found that the difference of the mechanical properties in unsterile and sterile conditions was relatively small for most of the investigated specimens. In general, the maximum loads are higher for the unsterile meshes compared to the sterilized specimens. The most significant effect was observed for polyester sterilized at 100 °C (Table 1). The polyethylene specimen could not be tested at 121 and 134 °C due to significant shrinkage effects. However, a sterilization at 100 °C led to a small reduction of the maximum load (approximately 2.5% for the arithmetic mean value, Table 2).

It seems that 100 °C sterilization has a deeper impact on lactate production, LDH cytotoxicity test, and moreover, there is a decrease of the maximum tensile force. We neither have an explanation for our results nor have we found an association for this effect at 100 °C in our literature research.

The biocompatibility of the fibroblasts also changed during our investigations due to sterilization, both for the polyethylene LCMs and the polyester LCMs. Thus, all sterilized LCMs showed the lowest mitochondrial activity as a sign of cell death compared to the unsterilized meshes. The cytotoxicity (LDH measurement) was also lowest in the unsterilized meshes, while it increased significantly after steam sterilization, particularly in the case of polyester LCMs. Similarly, cell metabolism showed a greater drop in pH and an increase in lactate in the sterilized meshes. This corresponds to the results already published by Broll et al. in 2002 [22]. They showed in vitro experiments with human fibroblasts that resterilized polypropylene meshes after steam sterilization at 121 °C showed both a significant decrease in the proliferation index and a significant increase in the apoptosis rate of the fibroblasts compared to the control and the unsterilized meshes [22]. As in our experiments, the sterilization process changes the growth behavior of the cells. The authors assume that the thermal treatment of the meshes damages the DNA and conclude that a malignant transformation of the tissue surrounding the sterilized meshes is possible over years or decades and could lead to the induction of sarcomas [22].

Mitura et al. also showed that massive shrinkage of mosquito meshes can occur [14]. The authors also carried out chemical and mechanical tests on the various LCMs and found massive changes (shrinkage, deformation) in meshes from Ethiopia, Ghana and India after sterilization at 121 and 134 °C [14]. Mitura et al. clearly stated that the chemical composition of the locally acquired meshes is not known, so that a certain risk is present and therefore the unrestricted use of LCMs cannot be recommended [14]. For the local producers of mosquito meshes, the suppliers of the raw materials can change every year, e.g. for cost reasons, so that an externally identical mesh can now change significantly during steam sterilization due to the change in composition. This is no problem for the producers of LCMs—they do not produce their nets for use as medical devices in humans! Löfgren et al. have also recommended that the mesh used in a randomized prospective study should no longer be used in its current form [23]. A general use of LCMs, as recommended in the current HerniaSurge Guideline, is, despite all the known economic problems in LICs, only recommended with very severe limitations and should be critically reviewed.

In our view, two approaches should be pursued.

On the one hand, the training of local surgeons, especially for suture-based procedures, should be intensively promoted. For example, Mitura et al. showed that there are anatomical differences in the inguinal region between Africans and Caucasians and that therefore pure tissue repairs could be promising, especially for African patients [24]. A recent Cochrane analysis also recommends mesh-free methods for LICs [25].

Since only suture-based procedures are not always feasible for the very large inguinal hernia gaps, which are often very common, and thus meshes are urgently required, we also see, like Löfgren et. al., a possibility to solve the problem by establishing a manufacturing facility in Central Africa [23].

## Conclusion

The sterilization of LCMs leads to significant changes in the growth behavior of human fibroblasts in vitro as well as in the chemical and mechanical properties of the meshes. Since manufacturers of LCMs do not produce certified medical devices, the chemical composition of meshes that have already been used clinically for positive results can change practically every day, so that for ethical (and legal) reasons alone a general use of LCMs cannot be recommended. A change to the, albeit, weak recommendation in the HerniaSurge Guideline is urgently recommended.
